# Phenotype forecasting with SNPs data through gene-based Bayesian networks

**DOI:** 10.1186/1471-2105-10-S2-S7

**Published:** 2009-02-05

**Authors:** Alberto Malovini, Angelo Nuzzo, Fulvia Ferrazzi, Annibale A Puca, Riccardo Bellazzi

**Affiliations:** 1 IRCCS Multimedica, Via Fantoli 16/15, I-20138 Milano, Italy; 2 Department of Computer Engineering and Systems Science, University of Pavia, via Ferrata 1, I-27100, Pavia, Italy

## Abstract

**Background:**

Bayesian networks are powerful instruments to learn genetic models from association studies data. They are able to derive the existing correlation between genetic markers and phenotypic traits and, at the same time, to find the relationships between the markers themselves. However, learning Bayesian networks is often non-trivial due to the high number of variables to be taken into account in the model with respect to the instances of the dataset. Therefore, it becomes very interesting to use an abstraction of the variable space that suitably reduces its dimensionality without losing information. In this paper we present a new strategy to achieve this goal by mapping the SNPs related to the same gene to one meta-variable. In order to assign states to the meta-variables we employ an approach based on classification trees.

**Results:**

We applied our approach to data coming from a genome-wide scan on 288 individuals affected by arterial hypertension and 271 nonagenarians without history of hypertension. After pre-processing, we focused on a subset of 24 SNPs. We compared the performance of the proposed approach with the Bayesian network learned with SNPs as variables and with the network learned with haplotypes as meta-variables. The results were obtained by running a hold-out experiment five times. The mean accuracy of the new method was 64.28%, while the mean accuracy of the SNPs network was 58.99% and the mean accuracy of the haplotype network was 54.57%.

**Conclusion:**

The new approach presented in this paper is able to derive a gene-based predictive model based on SNPs data. Such model is more parsimonious than the one based on single SNPs, while preserving the capability of highlighting predictive SNPs configurations. The prediction performance of this approach was consistently superior to the SNP-based and the haplotype-based one in all the test sets of the evaluation procedure. The method can be then considered as an alternative way to analyze the data coming from association studies.

## Background

Genetic association studies are a powerful method to assess correlations between genetic variants and traits differences occurring in a population. When a significant correlation arises with respect to a pathological trait, these studies may lead to the identification of candidate disease susceptibility genes, offering the promise of novel targets for therapeutic treatments. Nowadays, high-throughput genotype technologies allow a genome wide approach to these studies, taking into account hundreds of thousands of different markers [1,2]. Standard statistics is usually applied to this data to extract univariate models and find significant markers with univariate tests. However, together with deriving the existing correlation between genetic markers and phenotypic traits it is also extremely interesting to find the relations between the markers themselves. Both aims can be effectively achieved by using Bayesian networks (BNs) [3]. BNs represent probabilistic relationships between random variables by means of a directed acyclic graph and a set of conditional probability distributions. Nodes in the graph correspond to variables and directed arcs represent dependencies between them. A conditional probability distribution is associated with each node and quantifies the dependency of the node on its parents, i.e. the nodes that have an arc directly pointing to it.

BNs have already been successfully applied in association studies, for example to study overt stroke in sickle cell anaemia [4] and to identify the relationships between SNP variations in the human *APOE *gene and plasma apolipoprotein E levels [5].

**Figure 1:**
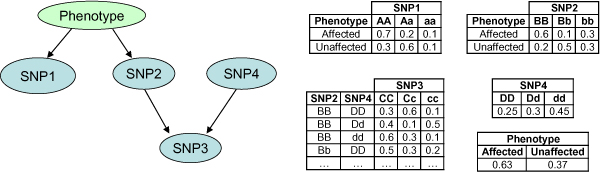
**Example of Bayesian network representing the dependencies between a Phenotype and 4 SNPs**. On the left, the directed acyclic graph of the BN; on the right the conditional probabilities tables associated with each node.

When performing an association study, the data typically consist of measurements for a set of genetic markers (SNPs) and evidence for a certain number of phenotypic traits (such as disease status, age, sex...). Each genetic marker is modelled as a random variable taking on one of three possible states: 'AA', which corresponds to homozygous for the minor allele, 'Aa', heterozygous, and 'aa', homozygous for the major allele. Each phenotypic trait is also represented by a random variable, such as 'affected' and 'unaffected' for the disease status. An example of BN modelling the relationships between 4 SNPs and a phenotypic trait is given in Figure 1. This network not only models the relationships between the phenotype and SNPs, but it also represents conditional independence assumptions between variables. Referring to the Figure, we can for example say that the phenotype is conditionally independent of SNP_3 _and SNP_4 _given SNP_2_: this means that, if the value for SNP_2 _is known, the phenotypic status does not depend on the values of SNP_3 _and SNP_4_. Thus, the BN can highlight potential key markers in phenotype prediction.

Both the graphical structure of a BN and the parameters of the conditional probability distributions can be learned from the available data. However, learning these networks is often non-trivial due to the high number of variables to be taken into account in the model, with respect to the instances of the dataset. Therefore, it becomes very interesting to use an abstraction of the variable space that suitably reduces its dimensionality without losing information. Thanks to this abstraction, a more parsimonious model might be built, whose graphical connections are also more easily interpretable. As the final aim of genetic dissection studies is to identify how genes affect the phenotype, we decided to consider the set of SNPs mapping to the same gene as a new meta-variable. In order to assign states to the meta-variables we employed an approach based on classification trees. By learning a classification tree for the SNPs mapping to each gene, it is possible to identify the most relevant combination of SNP values to predict the phenotypic status. Once the meta-variables have been identified, a BN is built using them and the phenotype as nodes.

We applied our method to genotypic data measured in a group of patients affected by arterial hypertension and in a group of nonagenarians without history of hypertension. The ability of the BN inferred on the meta-variables to correctly predict the phenotype (hypertension) is quantitatively assessed and compared with that achievable with a BN built using single SNPs.

## Methods

Our goal is to build a model to estimate the probability of a phenotypic trait given the genotype of an individual, represented as a suitable collection of SNPs. When learning this model from data, we also want to extract the relationships between SNPs and highlight the potential role of the genes associated to the SNPs. To this end, it is possible to resort to classification algorithms, in which the phenotype is the class and the SNPs (and potentially other interesting variables, such as sex and age) are the predictive attributes.

Our strategy is made of two main steps: i) generation of meta-variables corresponding to each gene by using an approach based on classification trees, ii) learning of a BN in which the nodes are the meta-variables and the phenotype.

**Figure 2:**
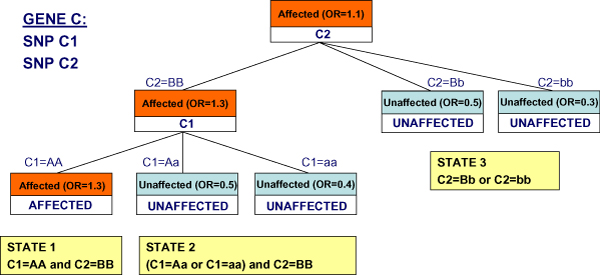
**Classification tree for meta-variable state assignment**. Example of classification tree used to infer the possible states of the meta-variable associated with gene C, represented by two SNPs, C1 and C2. OR = "Odds Ratio".

Classification trees (CTs) are one of the most largely used classification tools [6]. Given a database of *n *cases, each containing the values for *v *attributes and a class *c*, a CT learned from this database graphically represents a set of rules that allow the classification of each case on the basis of its attribute values (Figure 2). A test on the value of an attribute is associated with every non-leaf node of the tree and a branch descends from this node for every possible value taken by the attribute; leaf nodes are instead associated with a class value. Therefore the path going from the root node to a leaf node can be mapped into a classification rule of the kind "*if *attribute_A = a_1 _and attribute_B = b_2 _and attribute_C = c_1 _*then *class = c_i_".

Bayesian networks [7] are a formalism for the representation and use of probabilistic knowledge widely employed in various fields, such as Artificial Intelligence, Statistics, and more recently Bioinformatics. As mentioned in the Background Section, a BN consists of two main components, a directed acyclic graph and a set of probability distributions. While the graph qualitatively describes dependence relationships between variables, a conditional probability distribution is associated with each node X_i _and quantifies the probabilistic dependence of the node on its parents pa(X_i_). A very interesting property of BNs is the fact that the joint probability distribution of all variables can be expressed as the product of these conditional distributions (chain rule): $P(X_{1},...,X_{n})=\prod_{i}P(X_{i}|pa(X_{i}))$. Once a BN is learned it is possible to use it to perform probabilistic inference, i.e. to calculate the posterior probabilities of unobserved variables on the basis of evidence on the values of other variables in the network [8]. A BN can thus be employed for classification purposes, allowing the prediction of the most probable value for a class node once the values of some attributes are known.

In the following of this section we describe how we employ CTs to generate meta-variables and how we learn BNs on the generated variables.

### Meta-variables generation

There are different available algorithms to learn a classification tree from a dataset. Partitioning algorithms recursively split the tree by choosing the "most informative" attribute, i.e. the attribute that better separates instances with respect to their class value. These algorithms usually implement some "pruning" strategies, i.e. they remove leaves corresponding to negligible improvements in the classification performance. Pruning helps avoiding overfitting and thus helps improving the tree's ability to classify new instances not used to generate the tree.

CTs allow us to find rules to assign state values to meta-variables. Our procedure is performed with the following steps:

1. Select the set of SNPs S_*i *_mapping to the *i*-th gene (see "Data collection and pre-processing" section for SNPs annotation details).

2. Learn a classification tree using the phenotype to be forecast as class and the set S_*i *_as attributes. To this aim, we employed the C4.5 algorithm [9].

3. Prune the tree according to the following rules:

a. Apply minimal error pruning with m-estimate [10] and equal prior probability for each class. In the following all the results have been obtained with m = 8.

b. Remove leaves containing a number of instances lower than 1% of the total number of individuals (in our case, 5 instances).

c. Check the total number of meta-variable states that remain after pruning steps a and b: if there are more than 5 states, cut the subtree with the lowest number of instances.

4. Create a discrete variable G_*i *_with states corresponding to the leaves of the final pruned tree.

As an example, suppose having a gene C represented by two SNPs (C1 and C2), each taking three possible values ("AA" and "BB" stand for homozygous for the minor allele, "Aa" and "Bb" stand for heterozygous, "aa" and "bb" for homozygous for the major allele). Suppose also that the classification tree corresponding to gene C is shown in Figure 2.

Looking at the leaf nodes we can derive three rules to assign state values to the meta-variable for gene C, each one dependent on combinations of the two SNPs values: *if *"C1 = AA and C2 = BB" *then *State1; *if *"(C1 = Aa or C1 = aa) and C2 = BB" *then *State2; *if *"C2 = Bb or C2 = bb" *then *State3.

The classification trees were learned using the software Orange [11].

### BN learning

Learning BNs can be approached as a model selection problem, in which different network models are compared on the basis of their posterior probability with respect to the available data. Thanks to the decomposability of the joint probability of all variables, the network with highest posterior can be learned by learning local models, i.e. the parent sets of each variable, and then joining the inferred models. However, the number of possible models to be explored grows exponentially with respect to the number of candidate parents. For this reason, an exhaustive search is unfeasible and a heuristic strategy must be employed. An effective one is the greedy search strategy known as K2 algorithm [12]. This algorithm requires the specification of an ordering of the analyzed variables, so that the parents of each variable are searched only among those variables that precede it in the ordering. We decided to use the gain ratio of variables (i.e. the information gain divided by the variable's entropy [6]) to establish the ordering to be given as input to the K2 algorithm. In this way, variables with higher gain ratio were tested as parents of those with lower ratios. Moreover, we focused on networks in which the genotypes are dependent on the phenotype, in accordance with Sebastiani et al. [4].

In order to infer BNs from data we employed the software Bayesware Discoverer [13], which implements the K2 algorithm for the search.

## Results and discussion

We applied our approach to data coming from a genome-wide scan on 288 individuals affected by arterial hypertension (AH) and 271 nonagenarians without history of AH. Arterial hypertension is considered a polygenic disease, resulting from the combination of a number of genetic risk factors, whose expression depends on their interaction with environmental factors such as high dietary intake of sodium, alcohol, obesity and stress [14].

The number of alleles and polygenes contributing to the phenotype of elevated arterial blood pressure (BP) is still unknown; however, experiments in inbred rats suggest that about ten or more genes might contribute to the control of BP [15]. Moreover, although the number of genes influencing BP is not known, it is expected that many alleles at different loci may contribute to the ultimate disease trait. In agreement with these observations, linkage and association analyses have shown that BP is not due to a single genetic variant [15]. Our multivariate method thus appears particularly suitable to analyze this kind of data. In the following we describe in more details data collection, pre-processing and obtained results.

### Data collection and pre-processing

288 patients with high BP and aged 35–55 years were recruited; the control population was represented by 271 nonagenarians, without history of AH and selected during the course of the last few years. After approval of the ethical committee and under informed consent collected following the Italian law, blood was drawn from every patient participating in the study. DNA was extracted and anamnestic, clinical and laboratory data were collected. All samples were assayed with the Illumina HumanHap300Duo bead chips (Illumina, San Diego, CA, USA) containing 318,237 Phase I HapMap tagging SNPs. Data were acquired with Illumina Bead Studio Software (Illumina); afterwards, standard preliminary analysis was performed with gPLINK [16] as follows: i) genotyping/missing rate statistics were calculated; ii) the minor allele frequency (MAF) was calculated; iii) Hardy Weinberg Equilibrium (HWE) was evaluated; iv) SNPs with HWE values in the control population deviating from the equilibrium (p-value < 0.001) were removed. In order to identify and remove outliers, we performed a multidimensional scaling plot (MDS plot) and a neighbors plot, based on the genome-wide identity-by-state (IBS) information.

After data pre-processing, we performed both allelic and genotypic association tests to compare frequencies distribution between cases and controls and identify the most significant SNPs. Allelic association tests yielded 93 highly significant SNPs (p < 10^-4^, corrected for permutation tests). P-values given by genotypic association tests confirmed the same results as the allelic association tests.

SNP annotation was performed using *Genephony*, an online tool for genomic dataset annotation [17]: a SNP is annotated to a gene if it is located in a 10 Kb region around the coding sequence. Selecting only those genes represented by at least two SNPs, we focused on a subset of 24 SNPs mapping to 9 different genes. Thus the final dataset to be analyzed consisted of 559 individuals (288 cases and 271 controls) and the 24 selected SNPs. Since such results still have to be biologically validated, in the following we will denote genes by letters and SNPs by numbers, so that for example "D3" represents SNP number 3 of gene D.

### Bayesian networks for arterial hypertension prediction

**Figure 3:**
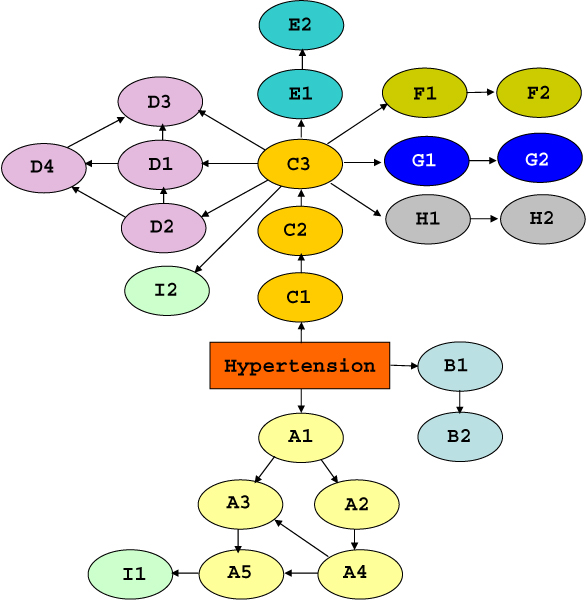
**SNP-based BN learned using the whole dataset**. Bayesian network learned on the whole dataset using single SNPs as variables.

We first learned a Bayesian network using single SNPs as variables and employing the whole dataset. In this network SNPs within each gene appear tightly connected (Figure 3). This is probably due to the fact that SNPs mapping to the same gene present highly correlated configurations and thus the BN learning algorithm correctly infers a direct dependence between them. This result supports the hypothesis that considering the SNPs mapping to the same gene as a unique meta-variable is an appropriate way to make an abstraction of the network structure without losing information. Hypertension is connected to 3 genes, and, among the SNPs within each gene, it is always directly connected to the SNP with the largest gain ratio.

**Figure 4:**
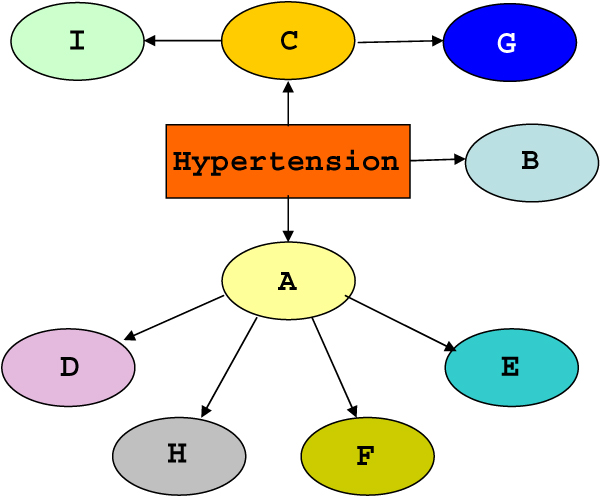
**Meta-variable BN learned using the whole dataset**. Bayesian network learned on the whole dataset using meta-variables associated to each gene.

We then associated each gene to a meta-variable, whose states were derived by building a classification tree according to the procedure outlined in the Methods section. In the network built using the meta-variables (Figure 4) the phenotype is directly connected to the same genes as in the network learned with all SNPs. Moreover the indirect path gene A – hypertension – gene C – gene G, identifiable in the single-SNP network, is conserved in the meta-variable BN. Therefore, the use of meta-variables appears able to summarize the relationships between genes and phenotype with little loss of information.

### Predictive ability of the networks

Bayesian networks allow the prediction of the most likely value for any node given the values of any group of other nodes. In our case, we are interested in assessing the ability of the learned BN models to predict the phenotype status given a certain configuration of SNPs or meta-variables.

The single-SNP network (Figure 3) and the meta-variable network (Figure 4) built on the whole dataset have a predictive accuracy (training accuracy) equal to 62.79% and to 64.22%, respectively. We compared these values with respect to the majority classifier performance, which had a classification accuracy of 51.52%. A normalized measure of how much those accuracies differ from the majority classifier is given by the k-statistic, which is 0.1541 for the SNP network and 0.2632 for the meta-variable one. Thus, the ability to predict the true phenotypic status of the meta-variable network appears to be slightly superior. However, as training accuracy is affected by overfitting, it is much more interesting to evaluate the capability of the models to correctly predict the unknown phenotype of a new sample of data (generalization accuracy). In order to obtain an estimate of the generalization accuracy, we repeated 5 times a random sampling hold-out scheme in which 75% of the dataset (419 individuals) was employed as training set and the remaining 25% as test set (140 individuals). The sampling was performed with stratification, so that both the training and the test set have the same distribution of phenotypic classes as the entire dataset. Thus, the validation scheme we performed 5 times can be summarized as:

• Split the dataset into 75% training and 25% test

• On the training set:

◦ Learn the meta-variables according to the strategy presented in the Methods section;

◦ Learn a BN using the gain ratio of meta-variables as ordering for the K2 algorithm.

• On the test set:

◦ Map the SNPs of the test set into meta-variables and assign the states using the rules derived on the training set;

◦ Compute the accuracy of phenotype prediction given all the meta-variables.

The mean accuracy on the 5 test sets was equal to 64.28% (Table 1). As a benchmark for our method, we used the BN with SNPs as variables, learned and evaluated on the corresponding test set. The mean accuracy of the SNPs BNs was 58.99%. Moreover, as reported in Table 1, the accuracy of the meta-variable network was always higher than the accuracy of the SNP network on the same test set, suggesting that our method is able to achieve a better classification performance. The k-statistic computed on each test set is also higher for the meta-variable networks. To confirm these results, we computed the 95%-confidence intervals (CI) of the mean accuracy for the meta-variable networks and for the single-SNP BNs (Table 1). Although the two CIs partially overlap, the mean accuracy of the meta-variable network is higher than the upper bound of the CI for the SNP-network. In order to evaluate the difference between the two sets of accuracies values, we applied the Wilcoxon signed rank test as suggested by J. Demsar [18], obtaining a significant result (p < 0.05). This reinforces the evidence that the performance of the meta-variable BNs is superior to the single-SNP ones.

**Table 1:** Classification performance on the test sets.

**Model**	**SNP based**		**Meta-variable based**		**Haplotype based**		**Majority Classifier**
**Classification Accuracies (%) and K statistics**	**CA**	**K-stat**	**CA**	**K-stat**	**CA**	**K-stat**	**CA**
Sampling test 1	55.71	0.09	64.28	0.26	57.14	0.12	51.43
Sampling test 2	55	0.07	59.28	0.16	53.57	0.04	51.43
Sampling test 3	63.57	0.25	67.86	0.34	55	0.07	51.43
Sampling test 4	62.14	0.22	65.72	0.29	49.29	-0.04	51.43
Sampling test 5	58.57	0.15	64.28	0.26	57.85	0.13	51.43
**Mean values on test sets**	**58.99**	**0.16**	**64.28**	**0.26**	**54.57**	**0.06**	**51.43**
95% Confidence Interval	54.28–63.72		60.36–68.2		50.34–58.80		
Standard Deviation	3.8		3.16		3.4		
Standard Error	1.7		1.41		1.52		

The table summarizes the results obtained by repeating 5 times a random sampling hold-out scheme in which 75% of the dataset (216 affected and 203 unaffected individuals) was employed as training set and the remaining 25% as test set (72 affected and 68 unaffected individuals). In particular, the table shows the classification accuracies obtained on the test sets by the single-SNP BN, the meta-variable BN and the haplotype BN, the accuracies of the majority classifier and the k-statistics.

### Comparison with haplotype-based BN

A typical way of performing association analysis using aggregated variables instead of single SNPs is to group them into haplotype blocks. Thus, we compared our classification approach with a haplotype-based one, considering haplotypes as variables to learn the BN. Haplotype definition was performed through the following steps:

• linkage disequilibrium (LD) analyses using Haploview software [19] to identify haplotype blocks for each gene region;

• haplotype blocks filtering to keep only blocks with frequency in the dataset larger than 10% (the frequencies are estimated using the expectation-maximization algorithm [20]);

• selection of the most informative haplotype configuration for each haplotype block, according to a case-control analysis based on permutation tests on the whole dataset;

• inference of the haplotype phases for each individual on the previously selected blocks (we used PLINK software package [16]);

• removal of individuals with a posterior haplotype probability < 0.80.

**Figure 5:**
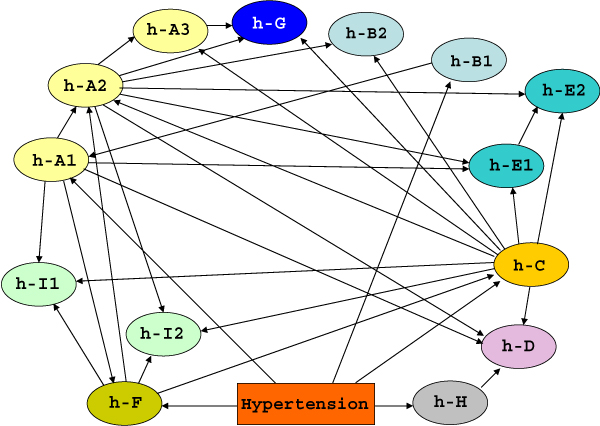
**Haplotype-based BN learned using the whole dataset**. Bayesian network learned on the whole dataset using haplotypes as variables.

After haplotype phases reconstruction and individuals selection, we learned a BN using haplotype phases as variables and we then applied the same validation scheme previously described for the single-SNP and meta-variable based approaches. The network built on the whole dataset is represented in Figure 5: the phenotype is connected to 5 haplotypes out of 14 (h-A1, h-B1, h-C, h-F and h-H), which map to 5 genes. Three of these genes (A, B and G) are directly connected to hypertension also in the meta-variable network. Yet haplotypes belonging to the same genes are not always connected with each other and the network shows a higher interconnectivity among variables than the meta-variable one. The classification accuracy of this network is 65.83% and thus higher than the single-SNP and meta-variable networks. However, the classification performances on the hold-out test sets are lower than the other two approaches, with an average classification accuracy of 54.57% (Table 1). The haplotype method seems prone to overfit the data, since it has the worst performance in terms of generalization accuracy, but the best one in terms of training accuracy. We also applied the Friedman statistical test [18] to verify the difference in the median accuracy of the three methods, obtaining significant results (p < 0.05).

## Conclusion

The new approach presented in this paper can be considered as an instrument to derive a gene-based predictive model based on SNPs data. Such model is more parsimonious than the one based on single SNPs, while preserving the capability of highlighting predictive SNPs configurations. Its limited number of nodes provides an abstract view of the relationships between genes and the phenotype of interest, and therefore represents an alternative way to analyze the available data. The prediction performance of this approach was consistently superior to the SNP-based and the haplotype-based one in all the sets studied in the paper.

The results of this approach should however be cautiously interpreted. First of all, the proposed learning method heavily exploits the training set, and, therefore, is prone to overfit the data. The evaluation should be performed on a separate test set to carefully assess the predictive performance. Moreover, learning the meta-nodes requires classification tree pruning to reduce overfitting. For this reason, the learning process needs to specify tree pruning parameters.

Furthermore, the method's goal is to perform prediction and the meta-variables have a precise meaning as predictors of a phenotype of interest. The method is designed to extract models based on gene-related SNPs, and cannot be properly applied to intergenic SNPs.

Finally, the results have been obtained after a selection of SNPs performed on the entire dataset. For this reason, the absolute values of the accuracies here reported are probably overestimated. However, since all the analyzed BNs have been learned with the same set of SNPs, their comparison is fair.

As regards the limits of the association study, we are aware of the potential confounding factors related to age and AH differences between groups. To address this issue we are genotyping an age-matched control population.

## Competing interests

The authors declare that they have no competing interests.

## Authors' contributions

AM carried out the molecular genetic studies, performed the statistical analysis and drafted the paper. AN carried out software tools development and integrations, participated in study design and drafted the manuscript. FF participated in the definition of the methodology and in drafting and reviewing the manuscript. AAP participated in study coordination and draft revision. RB conceived the study, participated in its design and coordination and helped to draft the manuscript. All authors read and approved the final manuscript.
